# Comparative Value of Zinc in Chorea

**Published:** 1860-07

**Authors:** 

**Affiliations:** Medical Registrar of St. Thomas’ Hospital


### COMPARATIVE VALUE OF ZINC IN CHOREA.

BY DR. STONE, MEDICAL REGISTRAR OF ST. THOMAS' HOSPITAL.

Dr. Stone's conclusions are based upon fifty cases of chorea admitted into St. Thomas' Hospital during the year 1858.

The general summary is as follows:

Of 16 cases treated with sulphate of zinc, 13 went out cured, 3 relieved; but two of the latter were in a fair way of recovery, and may probably be set to the credit of the medicament. On the other hand, three of those ultimately cured owed their improvement partly to ferruginous preparations, and in one case the zinc had no effect whatever. It may, then, be stated generally, that advantage was derived from the zinc in 14 out of 16 cases. The longest stay in the hospital among these cases, was 123 days; the shortest, 14; the average stay, 44.6 days.

Fourteen cases were treated during the same period with preparations of iron; all were cured. The longest stay in the hospital was 161 days; the shortest, 6 days; average stay, 44.2 days.

Twenty cases were treated with liq. potassæ arsenitis: 18 cured, 1 relieved, 1 died. The longest stay in hospital was 55 days; the shortest, 6; average stay, 26.3 days.

Average stay in hospital of the 50 cases submitted to three principal remedies, 37.2 days; average stay of all the 54 cases, 35.4 days.

The results of this analysis are somewhat remarkable, as failing to confirm the usual estimate of the value of sulphate of zinc in this disorder. The iron seems to act more certainly, and the arsenic both more certainly and more rapidly, than the zinc. The average duration of treatment, both with iron and zinc, 44.2 days and 44.6 days respectively, is very similar, and both are above the general average of the whole number of cases, namely, 35.4 days; whereas the average stay of the arsenic cases, falls as low as 26.3 days. This difference is the more remarkable, as the character of the cases submitted to the arsenical treatment rather exceeded in severity that of the other; and, indeed, the only death recorded belonged to this division.

It remains a question whether the discrepancy between these results and those of some previous well-conducted observations is due to mere accident, or to some real difference in type between cases originating at different times, and under dissimilar circumstances.

